# P-137. Optimizing Endocarditis Risk Assessment: Validation of the HANDOC Score for Non-Beta Hemolytic Streptococcal Bacteremia in a Mixed Practice Hospital Setting

**DOI:** 10.1093/ofid/ofaf695.364

**Published:** 2026-01-11

**Authors:** Perani V Chander, Brianna Desa, Vaishnavi Sirekulam, Bourann Husainy, Mohamed Elhussain, Anisha Pareddy, Ahmad Kofahi, Matthew T Brennan, Mazhar Shapoo, America Silva, Alex Huang, Hussein Tehaili, Lea M Monday

**Affiliations:** Wayne State University School of Medicine/Detroit Medical Center, Detroit, MI; Wayne State University School of Medicine/Detroit Medical Center, Detroit, MI; Wayne State University School of Medicine/Detroit Medical Center, Detroit, MI; Wayne State University School of Medicine/Detroit Medical Center, Detroit, MI; Wayne State University School of Medicine/Detroit Medical Center, Detroit, MI; Wayne State University School of Medicine/Detroit Medical Center, Detroit, MI; Wayne State University School of medicine, Detroit, Michigan; Wayne State University School of Medicine, Detroit, Michigan; Detroit Medical Center, Detroit, Michigan; Wayne State University School of medicine, Detroit, Michigan; Wayne State University School of Medicine/Detroit Medical Center, Detroit, MI; Wayne State University School of Medicine/Detroit Medical Center, Detroit, MI; Wayne state University School of Medicine, Detroit, Michigan

## Abstract

**Background:**

Non-β-hemolytic streptococci (NBHS) bacteremia is a cause of infective endocarditis (IE). The Duke criteria, while imperfect, are a cornerstone of standardization for the definition and diagnosis of IE. It is unfeasible to obtain a transesophageal echocardiography (TEE) on all NBHS bacteremic patients; The HANDOC score is a tool to identify patients at low risk of IE unlikely to need TEE, but its validation across patient populations is limited (Fig 1). We aimed to validate the HANDOC score in a hospital system with a mixed practice of academic and private groups and no dedicated IE team.

**Figure:**
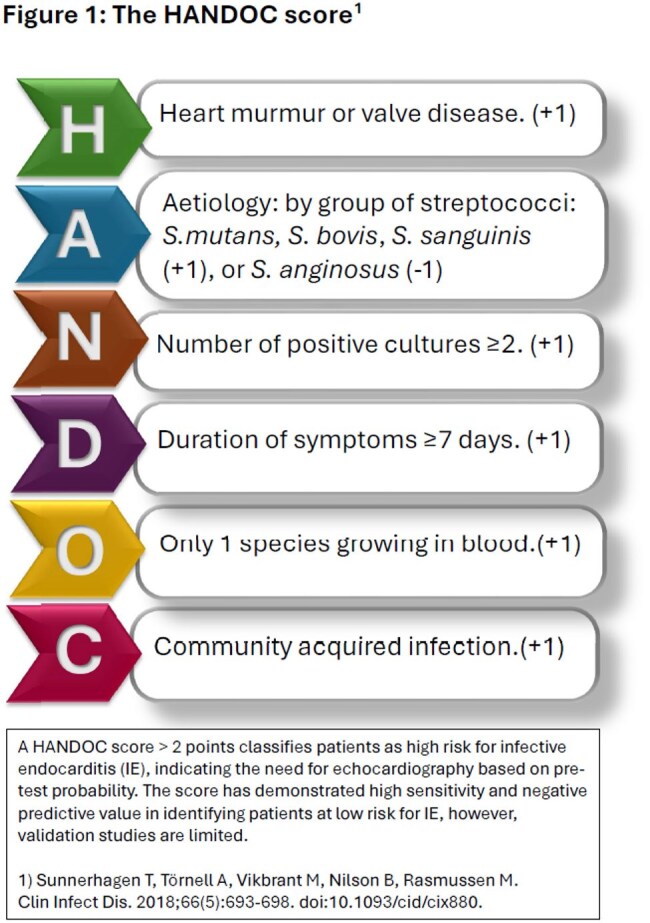

**Figure:**
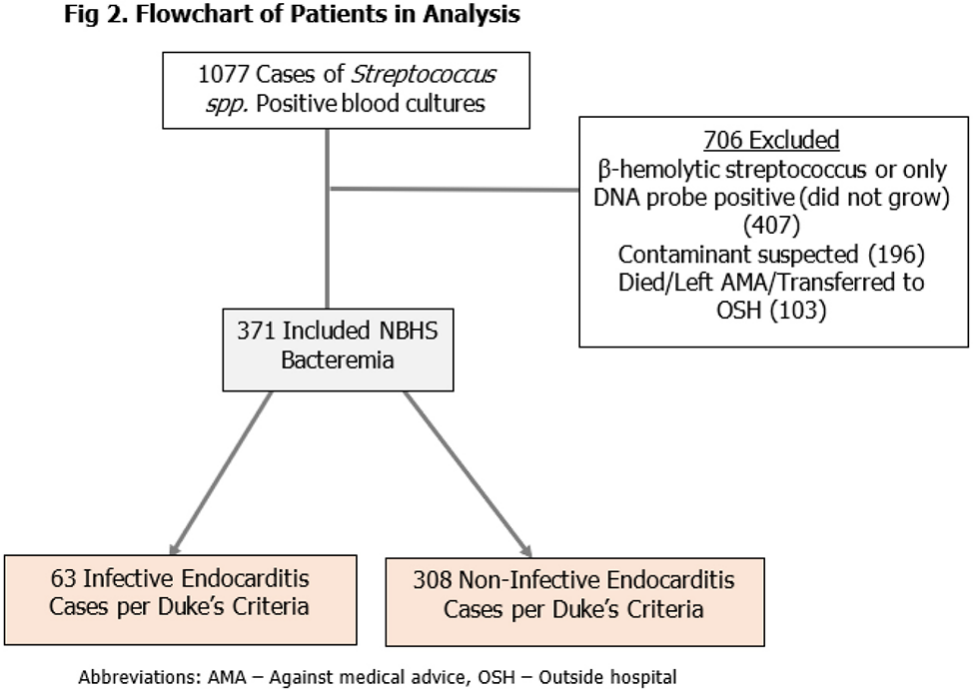

**Methods:**

This retrospective study enrolled all patients with NBHS bacteremia admitted to a safety net hospital system in Detroit, MI from 1/2021-10/2024. IE was defined per 2023 modified Duke Criteria for definite IE. Clinical and microbiologic characteristics were collected. Patients were excluded if NBHS was a suspected contaminant, NBHS did not grow on culture, or if they expired or left against medical advice (Fig 2).

**Figure:**
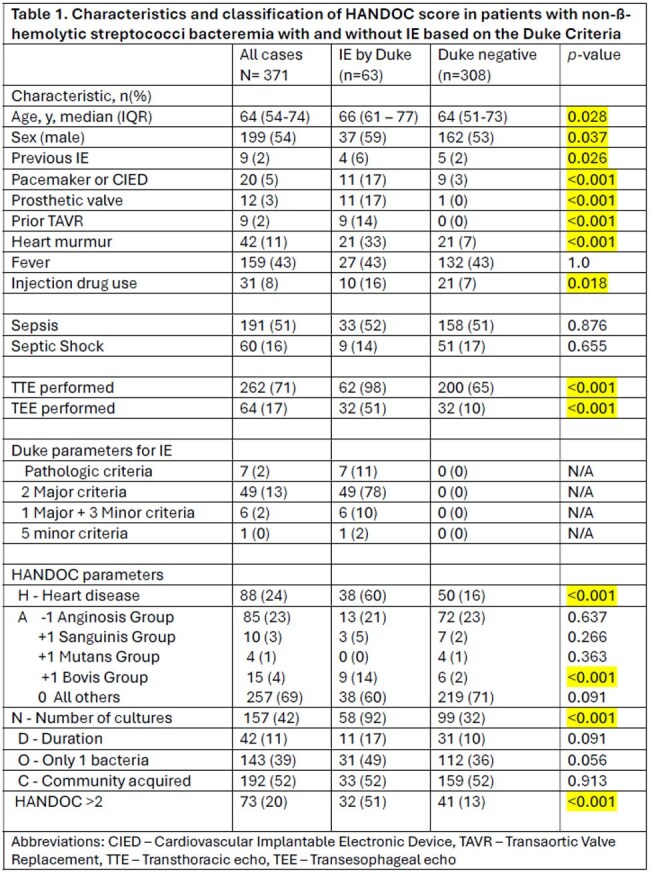

**Figure:**
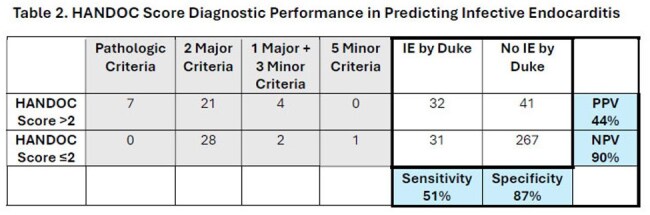

**Results:**

371 patients were included (63 with IE, and 308 without IE). IE patients were older with higher rates of cardiac conditions and drug use (Table 1). Sepsis parameters were similar between groups. The most common Duke criteria met for IE diagnosis was 2 major criteria (78%). Valve pathology was present in 11%. HANDOC score > 2 occurred in 73 patients (32 with IE, 41 without). The "H," "N," and *Bovis* group parameters were higher in the IE group, while community onset and other NBHS species were similar across groups. The HANDOC score had a NPV of 90% and specificity of 82% but a lower PPV and sensitivity (Table 2).

**Conclusion:**

Diagnostic stewardship in IE is challenging due to the limitations of the Duke criteria and lack of dedicated IE team in most hospital systems. A HANDOC score < 2 can be used by clinicians to safely forego the need for an echocardiogram in NBHS, thereby decreasing resource use and unnecessary invasive testing.

**Disclosures:**

All Authors: No reported disclosures

